# Preparation magnetic graphene oxide/diethylenetriamine composite for removal of methylene blue from aqueous solutions

**DOI:** 10.1038/s41598-024-65628-7

**Published:** 2024-07-04

**Authors:** Alireza Banaei, Afshin Saadat, Roghayyeh Javadi, Parinaz Pargolghasemi

**Affiliations:** 1 Department of Chemistry, Payame Noor University, P.O. Box 19395-3697, Tehran, Iran; 2 Department of Chemistry, Germi Branch, Islamic Azad University, Germi, Iran

**Keywords:** Green chemistry, Inorganic chemistry

## Abstract

Graphene oxide (GO) and its derivatives have several applications in many areas such as environmental and energy materials, water treatment and biomedical technologies. Because of having various polar groups on its surface, GO is considered as an excellent adsorbent. However, for many applications such as adsorption of pollution from aqueous solutions, chemical functionalization of graphene oxide is often a necessary requirement. In the present study, a new composite from graphene oxide, diethylenetriamine (DETA) and silica coated MnFe_2_O_4_ nanoparticles (GO/DETA/MnFe_2_O_4_@SiO_2_) was prepared. The structure, thermal stability and magnetic properties of the composite were studied by FT-IR, XRD, SEM, EDS, VSM and TGA spectroscopic methods. The prepared composite showed magnetic property with a saturation magnetization of 3.0 emu/g. The adsorption properties of GO/DETA/MnFe_2_O_4_@SiO_2_ composite for methylene blue (MB) in aqueous solution were studied using batch method. The effects of important parameters on the surface adsorption process of MB, including pH, contact time, adsorbent dosage and initial dye concentration were investigated. The adsorption isotherm was in accordance with Langmuir model showing surface homogeneity of the adsorbent. According to the Langmuir analysis, the maximum adsorption capacity (q_m_) of GO/DETA/MnFe_2_O_4_@SiO_2_ composite for MB was found to be 243.91 mg/g. The kinetic studies showed that the adsorption was pseudo first-order process. In addition, the thermodynamic studies indicated the adsorption was spontaneous and endothermic process.

## Introduction

In recent years, the growth of industries such as textile, leather, cosmetics and printing has increased pollution caused by dyes in water environments^1^. Organic dyes due to the toxic and carcinogenic are serious hazard to human, microorganisms and the ecosystem^2^. Therefore, it is urgent and important to detect and remove these toxic components from wastewater. Many methods such as adsorption^3^, electrochemical oxidation^4^, photocatalytic degradation^5^, ion exchange^6^ and nanofiltration membranes^7^ have been applied to remove dyes from wastewater. Among these methods, the adsorption process seems to be the ideal choice due to the low initial cost, easy design, suitable flexibility and high efficiency^8^. In recent years, many adsorbents such as zeolites^9^, activated carbon^10^, alumina^11^, silica gel^12^, bentonite clays^13^, etc. have been used for removal of toxic dyes from aqueous solutions.

One of the most important dyes used in the textile industry is methylene blue (MB), which is chemically called 3,7-bis(dimethylamino) phenazathionium chloride or tetra methylthionine chloride and is a lasting cationic dye^14^. MB is a dangerous, toxic and carcinogenic organic dye, whose release in water harms human health and the ecosystem^15^. Therefore, it is important to look for efficient methods and materials for removal of MB. A lot of different materials have been suggested as potential adsorbents for methylene blue, such as activated clay^16^, graphitic materials^17^, cauliflower leave^18^, garlic pee^19^, clay graphene oxide iron oxide^20^, layered double hydroxide polymer^21^, etc. However, the design and search for new adsorbents are still urgent to enhance the adsorption capacity and improve the separation rate for removal of MB from wastewater. Today, nanocomposites have a good potential to absorb MB due to their high surface area^22,23^. Nanocomposites consisting of two parts, organic and inorganic can play an effective role in improving adsorption^24^.

In this field, Graphene based nanocomposites have also been investigated for the removal of some organic dyes and MB from wastewater^25^. Graphene oxide (GO) has a special structure of a two-dimensional honeycomb lattice with a single layer of sp^2^ carbon atoms^26,27^. Studies show that GO is an effective adsorbent towards dyes and heavy metal ions^28–31^. Its electronic and characteristic structure supply the electrostatic force and π–π stacking effect, which facilitate and contribute to the adsorption process^32–35^. Furthermore, GO is composed of many functional groups such as carboxyl groups, hydroxyl and epoxide, which lead to a hydrophilic and negatively charged surface. GO can be simply functionalized applying different treatments to change the functional groups for the purpose of gaining the desired surface properties. GO derivatives especially those functionalized with heteroatom have shown better performance as compared to its counter parts of graphene oxide^36,37^. In this filed, GO-calcium/alginate nanocomposite was synthesized and used to adsorb MB by Li et al., and the adsorption capacity was 163.93 mg g^−1^ ^38^. Fan et al. synthesized a magnetic GO-chitosan nanocomposite and used it for removal of MB from aqueous solutions. The adsorption capacity was determined to be 95.31 mg g^−1^ ^39^. In another study, Dai et al. doped the synthetic polymers such as PVA (poly (vinyl alcohol)) with graphene oxide in order to increase the ability of adsorption of MB. The adsorption capacity of GO/PVA composite for MB removal was 127.5 mg g^−1^ ^40^.

**Figure 1:**
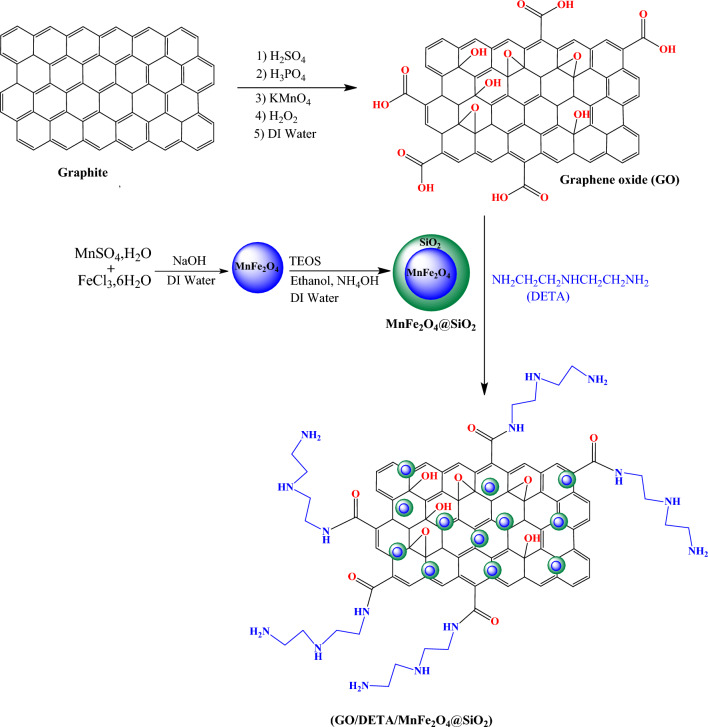
The synthesis route of GO/DETA/MnFe_2_O_4_@SiO_2_ composite.

In this study, a new composite from GO, diethylenetriamine (DETA) and silica coated MnFe_2_O_4_ nanoparticles (GO/DETA/MnFe_2_O_4_@SiO_2_) was synthesized (Fig. 1). The composite prepared was applied for the removal of the MB from aqueous solution. Moreover, the effects of various parameters such as pH, adsorbent dosage, initial dye concentration and contact time on adsorption behavior were studied. Adsorption isotherms, kinetics and thermodynamic studies have been reported to account for the nature of adsorption process.

## Results and discussion

### Characterization of GO/DETA/MnFe_2_O_4_@SiO_2_ composite

#### Infrared spectral characterization

**Figure 2:**
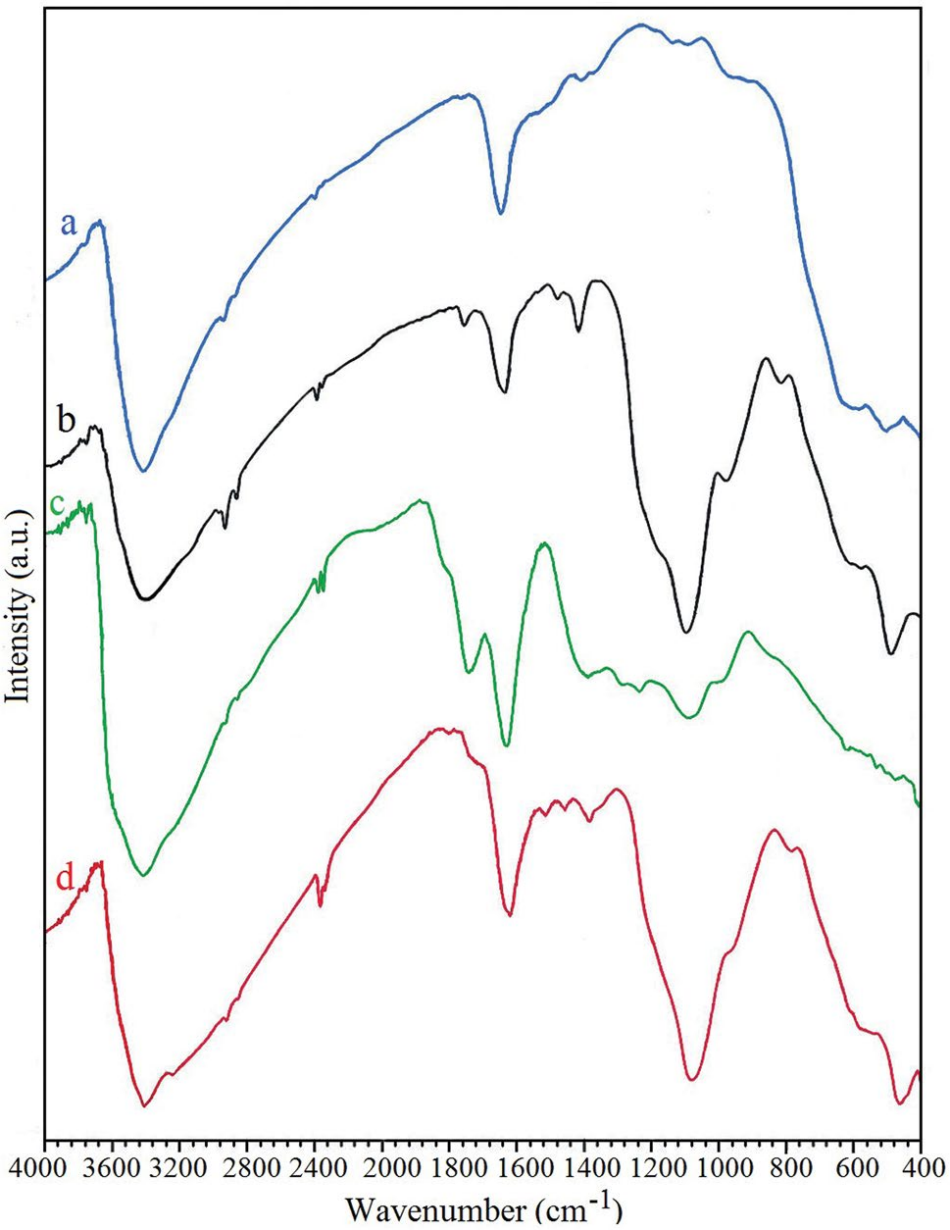
FT-IR spectra of (**a**) MnFe_2_O_4_, (**b**) MnFe_2_O_4_@SiO_2_, (**c**) GO, (**d**) GO/DETA/MnFe_2_O_4_@SiO_2_ composite.

The FT-IR spectra of synthesized compounds are demonstrated in Fig. 2. In MnFe_2_O_4_ FT-IR spectrum (Fig. 2a, blue line), a broad peak at 3428 cm^−1^ and a peak at 1627 cm^−1^ belong to stretching and bending vibration of hydroxyl (OH) groups, respectively^41^. Also, the peaks at 582 and 463 cm^−1^ are attributed to stretching vibration of Metal oxide (M–O) stretching vibrations of Mn–O and Fe–O, respectively^42^. The FT-IR spectrum of MnFe_2_O_4_@SiO_2_ nanoparticles is showed in Fig. 2b (black line). As it can be observed, MnFe_2_O_4_ nanoparticles after reaction with TEOS demonstrates a new peak at 1077 cm^−1^ which is assigned to stretching vibration of Si–O–Si. Figure 2c (green line) presents the FT-IR spectrum of GO. According to the GO FT-IR spectrum, a broad peak at 3600–2500 cm^−1^ belongs to stretching vibration of hydroxyl groups (OH) and adsorbed water molecules. Furthermore, the C=O stretching vibration of carboxylic acid group demonstrates a sharp peak at 1739 cm^−1^. Moreover, the peak at 1620 cm^−1^ is attributed to the stretching vibration mode of C=C band and the peaks at 1228 and 1052 cm^−1^ are attributed to the C–O stretching of phenolic and epoxy groups^43^. The FT-IR spectrum of GO/DETA/MnFe_2_O_4_@SiO_2_ composite is showed in Fig. 2d (red line). As it can be observed, after DETA and MnFe_2_O_4_@SiO_2_ modification, the peak of GO at 1739 cm^−1^ disappears indicating the reduction of C=O of carboxylic acid group because of amine functionalization. Also, new peaks appear at 1516 and 1085 cm^−1^ which belong to N–H bending and Si–O–Si stretching, respectively. Besides, in the FT-IR spectrum of GO the peak at 1381 cm^−1^ is attributed to C–OH band and due to ammination process its intensity has been decreased^44^.

### XRD analysis

**Figure 3:**
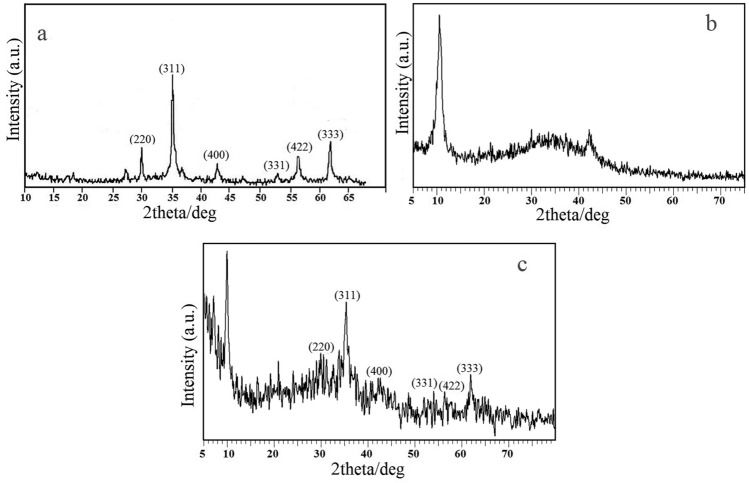
XRD pattern of (**a**) MnFe_2_O_4_ nanoparticles, (**b**) GO and (**c**) GO/DETA/MnFe_2_O_4_@SiO_2_ composite.

X-ray diffraction of MnFe_2_O_4_ nanoparticles, GO and GO/DETA/MnFe_2_O_4_@SiO_2_ composite are demonstrated in Fig. 3. For the MnFe_2_O_4_ nanoparticles, XRD patterns (Fig. 3a) show the typical peaks 2θ = 30.20°, 35.45°, 43.25°, 53.50°, 57.15° and 63.40°, which are in agreement with the referenced data for MnFe_2_O_4_ nanoparticles^45^. Figure 3b presents XRD patterns of GO. The strong diffraction peak at 2θ = 10.49° shows that interlayer spacing of GO based on the Bragg equation is 8.4 Å. Figure 3c shows XRD patterns of GO/DETA/MnFe_2_O_4_@SiO_2_ composite. The XRD pattern of GO/DETA/MnFe_2_O_4_@SiO_2_ composite includes the MnFe_2_O_4_ peaks with a peak at 2θ = 9.8° which is attributed to GO. The average particles size of GO/DETA/MnFe_2_O_4_@SiO_2_ composite is calculated via Debye–Scherer equation:

$$D = \frac{K\lambda }{{\beta \cos \theta }}$$

where D is the average size, λ is the X-ray source wavelength (1.54 Å), β is the full width at half maximum (FWHM) of the diffraction peak and θ is the Bragg’s angle.

The average particle size of the GO/DETA/MnFe_2_O_4_@SiO_2_ composite is obtained based on Debye–Scherrer equation 149 nm.

### SEM and EDS analysis

**Figure 4:**
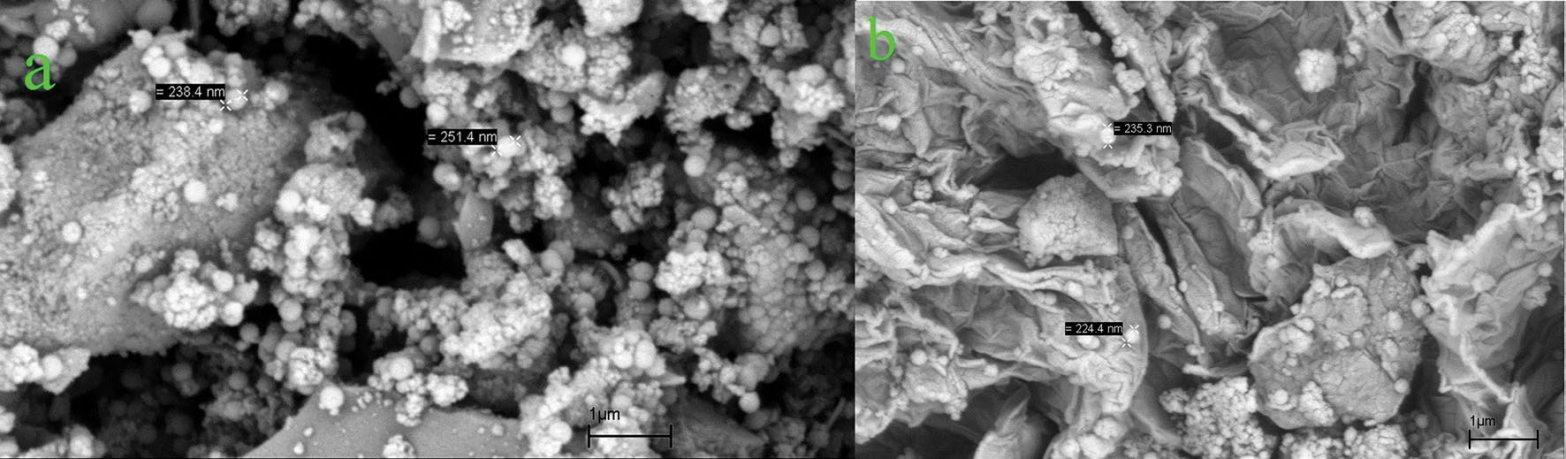
SEM images of (**a**) MnFe_2_O_4_@SiO_2_, (**b**) GO/DETA/MnFe_2_O_4_@SiO_2_ composite.

**Figure 5:**
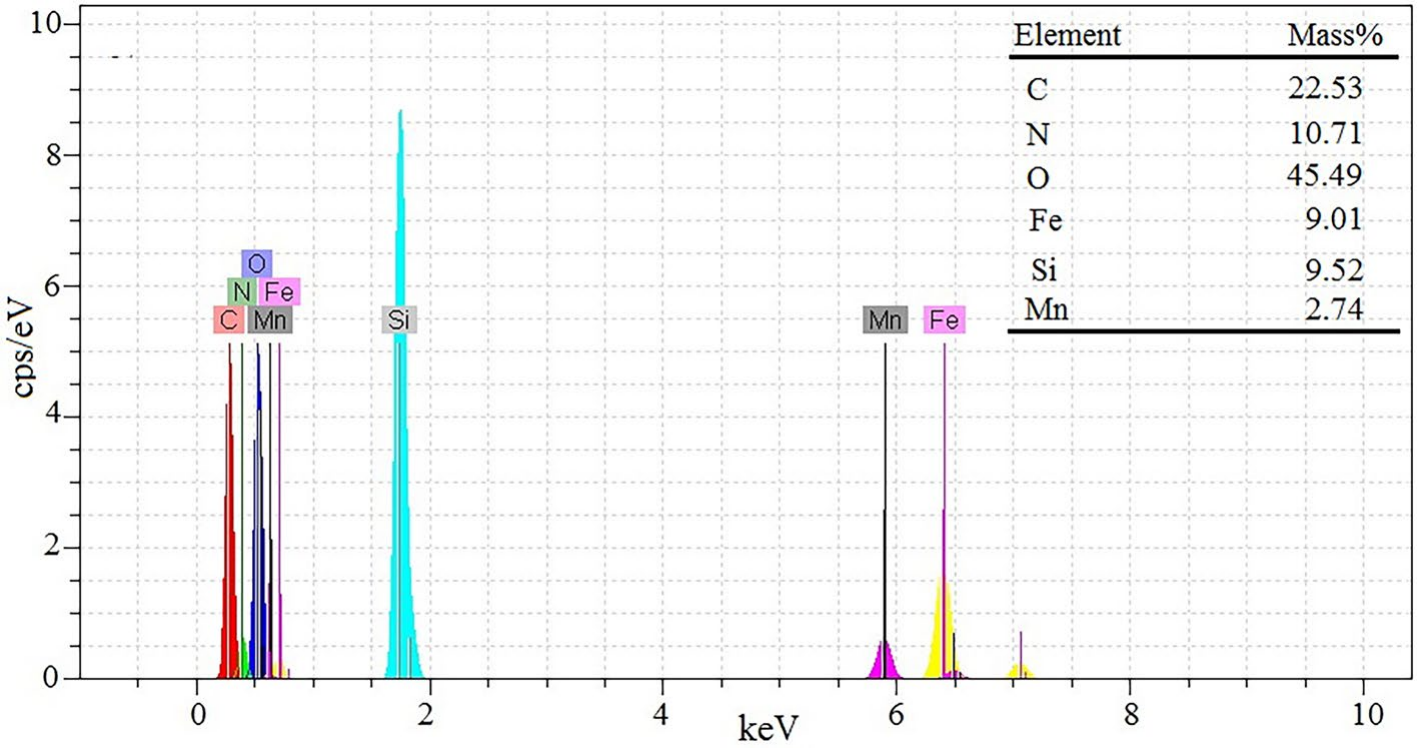
EDS patterns of GO/DETA/MnFe_2_O_4_@SiO_2_ composite.

The particle size and surface morphology of MnFe_2_O_4_@SiO_2_ and GO/DETA/MnFe_2_O_4_@SiO_2_ composite were studied applying SEM technique. As illustrated in Fig. 4a, MnFe_2_O_4_@SiO_2_ nanoparticles are mostly in spherical shape and have average particle size about 244 nm. Figure 4b demonstrates the SEM image of GO/DETA/MnFe_2_O_4_@SiO_2_ composite. SEM confirms verify the presence of spherical MnFe_2_O_4_@SiO_2_ nanoparticles on the surface of GO/DETA/MnFe_2_O_4_@SiO_2_ nanocomposite. The SEM image of GO/DETA/MnFe_2_O_4_@SiO_2_ composite obviously illustrates that it has created a layered structure and the spherical MnFe_2_O_4_@SiO_2_ nanoparticles have the average particle size about 230 nm. The chemical composition of GO/DETA/MnFe_2_O_4_@SiO_2_ composite is studied via EDS analysis. The EDS spectrum of composite is presented in Fig. 5. EDS measurement confirms that the composite contains C, N, O, Si, Mn and Fe. The EDS spectrum of GO/DETA/MnFe_2_O_4_@SiO_2_ composite shows the atomic percentage of C, N, O, Si, Mn and Fe are 22.53, 10.71, 45.49, 9.01, 9.52 and 2.74, respectively.

### Magnetization analysis (VSM)

**Figure 6:**
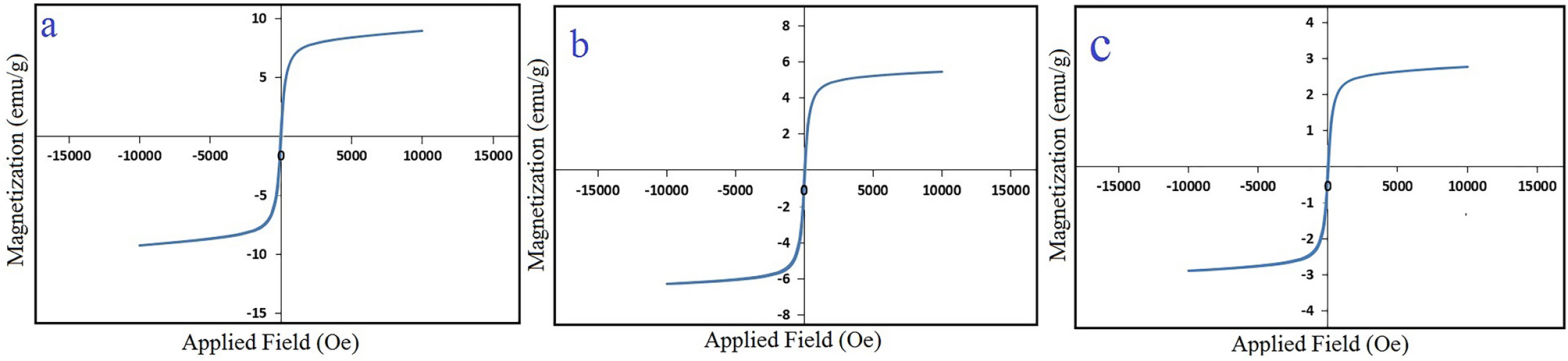
Magnetic hysteresis loops of (**a**) MnFe_2_O_4_, (**b**) MnFe_2_O_4_@SiO_2_ and (**c**) GO/DETA/MnFe_2_O_4_@SiO_2_ composite.

The magnetic moment of the MnFe_2_O_4_, MnFe_2_O_4_@SiO_2_ and GO/DETA/MnFe_2_O_4_@SiO_2_ composite were calculated over a range of applied fields between 10,000 and − 10,000 Oe. Figure 6 shows magnetization curves of the MnFe_2_O_4_, MnFe_2_O_4_@SiO_2_ and GO/DETA/MnFe_2_O_4_@SiO_2_ at room temperature. As presented, the saturation magnetization value of MnFe_2_O_4_ to GO/DETA/MnFe_2_O_4_@SiO_2_ is decreased sequentially. These results confirm that, the surface of the MnFe_2_O_4_ nanoparticles are successfully coated with silica, DETA and GO. The saturation magnetization values of MnFe_2_O_4_, MnFe_2_O_4_@SiO_2_ and GO/DETA/MnFe_2_O_4_@SiO_2_ are 10, 6 and 3 emu/g, respectively.

### TGA analysis

**Figure 7:**
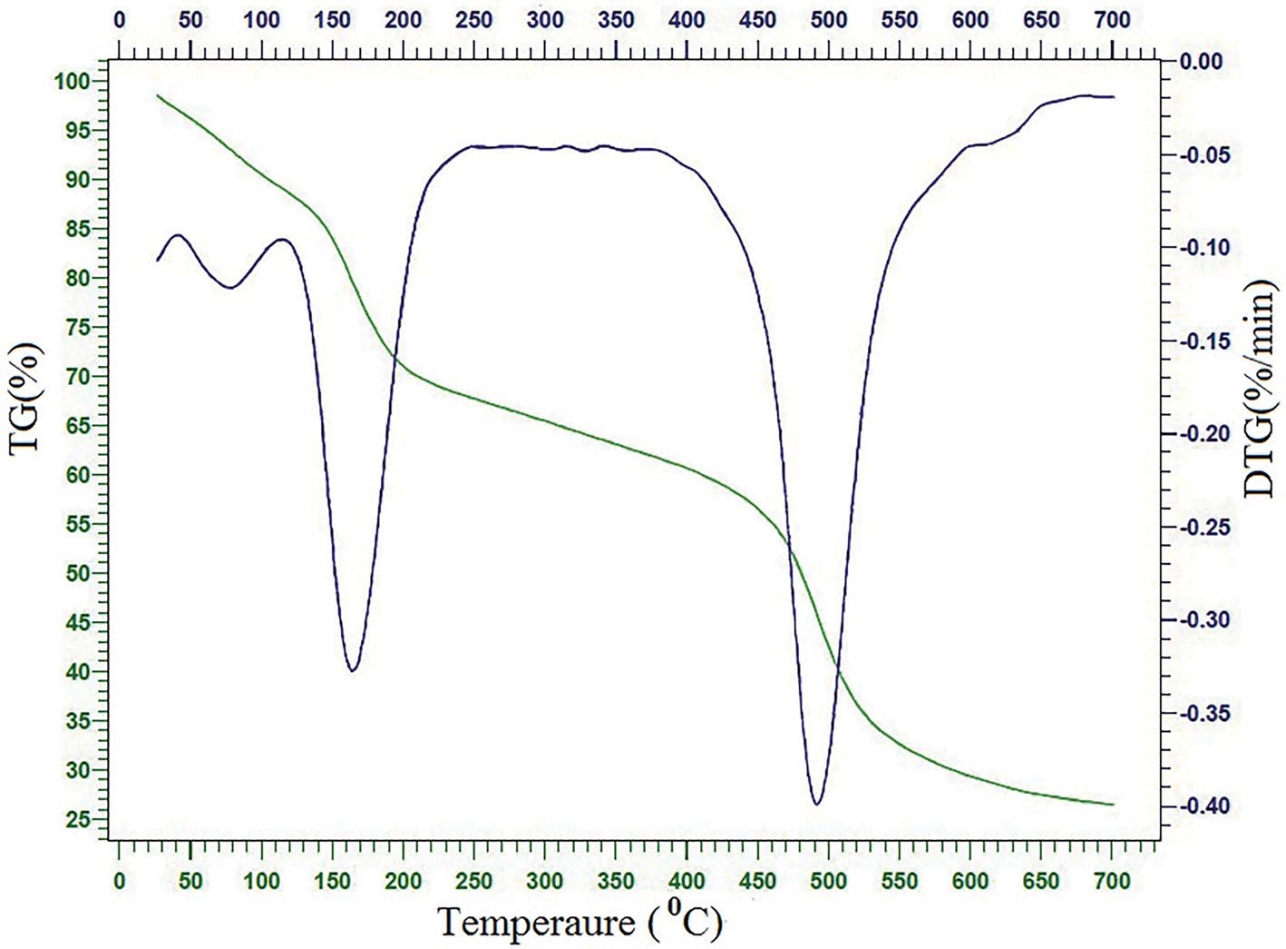
TGA-DTG curve of GO/DETA/MnFe_2_O_4_@SiO_2_ composite.

Thermo-gravimetric Analysis (TGA) was done to predict the thermal stability of the GO/DETA/MnFe_2_O_4_@SiO_2_ composite. Figure 7 demonstrates the TGA-DTG curve of the GO/DETA/MnFe_2_O_4_@SiO_2_ composite. The TGA-DTG revealed three-stage degradation pattern between 25 and 600 °C. The first stage degradation happened between 25 and 135 °C with 11.5%, which could be assigned to the release of adsorbed water in the sample^46^. The second weight loss (18.4%) within 135–250 °C can be related to the degradation of groups that contain oxygen^47^. In the third stage, in the range from 250 to 550 °C, the major weight loss occurred and was decomposed 34.7% of GO/DETA/MnFe_2_O_4_@SiO_2_ composite, which can be at attributed to the decomposition of the carbon skeletons of GO and DETA^48^.

### Sorption studies of selected dye

#### Effect of adsorbent dosage

**Figure 8:**
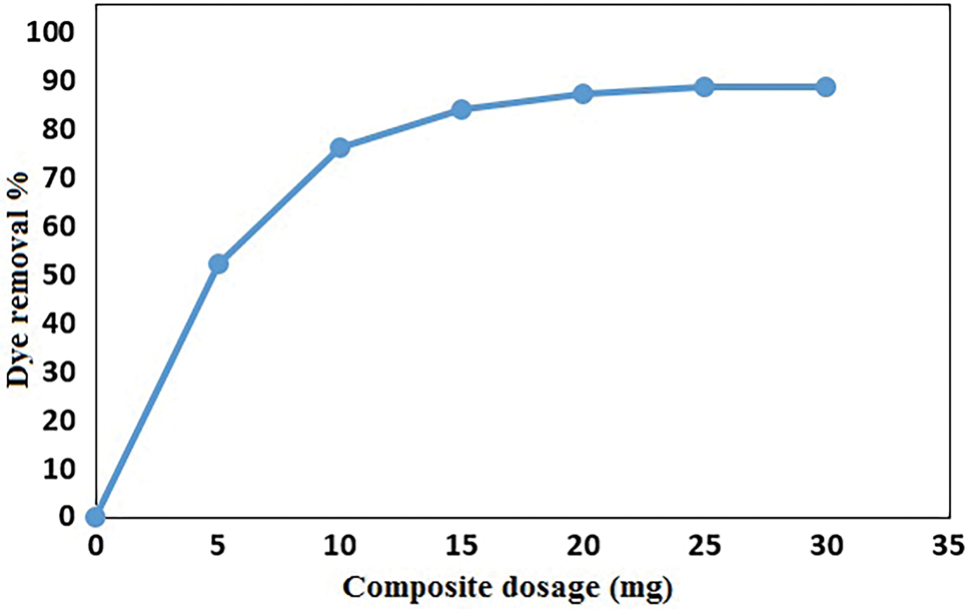
Effect of adsorbent dosage on removal of MB by GO/DETA/MnFe_2_O_4_@SiO_2_ composite (100 mg/l, 200 rpm, 25 °C, 10 min, pH = 7).

The important factor that influences adsorption processes is adsorbent dosage since it characterizes the capacity of adsorbent for a given initial concentration of dye solution^49^. In our work, the effect of adsorbent dosage on adsorption removal of MB was investigated with sorbent amounts in the range 0–30 mg in the adsorption systems containing 40 mL of 100 mg/l solution of dye at 25 °C for 10 min. Figure 8 displays the effect of adsorbent dosage on the percentage removal of MB. As shown, the percentage removal of MB increased from 0 to 89% with increasing GO/DETA/MnFe_2_O_4_@SiO_2_ composite. This phenomenon can be related to the increasing of the surface area of the adsorbent and availability of more adsorption sites. Based on the results in Fig. 8, an optimum adsorbent dosage of 25 mg was selected.

#### Effect of initial dye concentration

**Figure 9:**
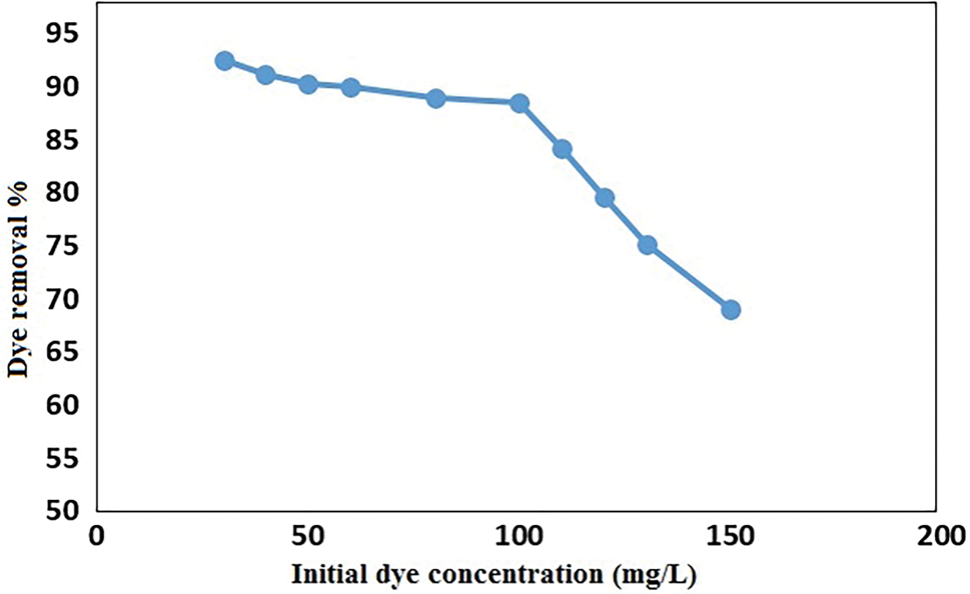
Effect of initial dye concentration on removal of MB by GO/DETA/MnFe_2_O_4_@SiO_2_ composite (25 mg, 200 rpm, 25 °C, 10 min, pH = 7).

The influence of initial dye concentration on adsorption in case of GO/DETA/MnFe_2_O_4_@SiO_2_ composite was studied with different solution concentrations (30–150 mg/l) applying 25 mg of adsorbent. As it is shown in Fig. 9, the dye removal percentage is decreased with the increase of the initial dye concentration, which may be due to the decrease of enough number of active sites of GO/DETA/MnFe_2_O_4_@SiO_2_ composite for binding on the dye molecules.

#### Effect of contact time

**Figure 10:**
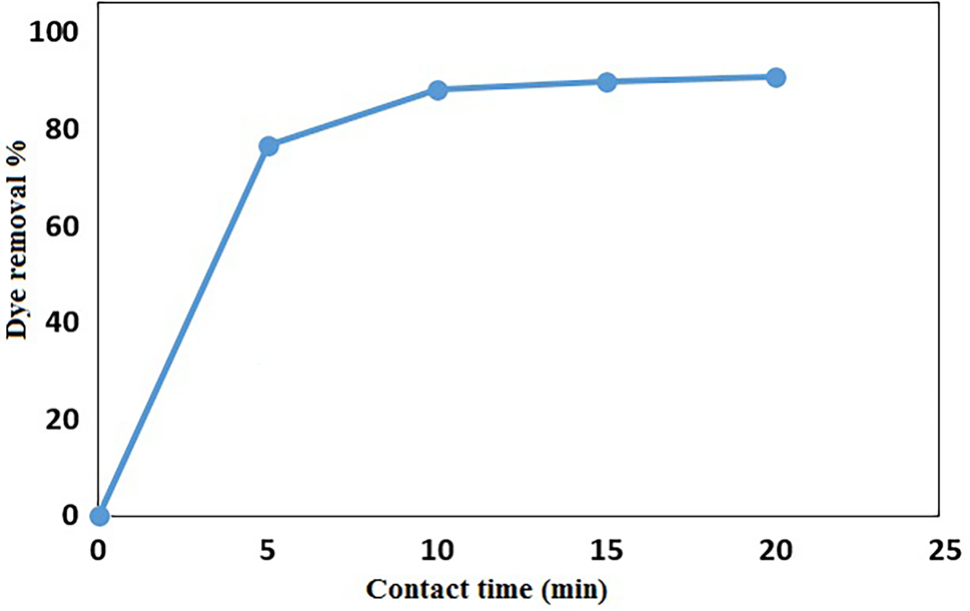
Effect of contact time on removal of MB by GO/DETA/MnFe_2_O_4_@SiO_2_ composite (100 mg/l, 25 mg, 200 rpm, 25 °C, pH = 7).

The effect of contact time on the adsorption of MB on GO/DETA/MnFe_2_O_4_@SiO_2_ composite is demonstrated in Fig. 10. As can be seen in Fig. 10, with increasing contact time, the adsorption percentage of MB has increased rapidly in the early stages because of high availability of vacant adsorption sites. After a period of 10 min, the adsorption illustrated a steady increase.

#### Effect of initial pH solution

**Figure 11:**
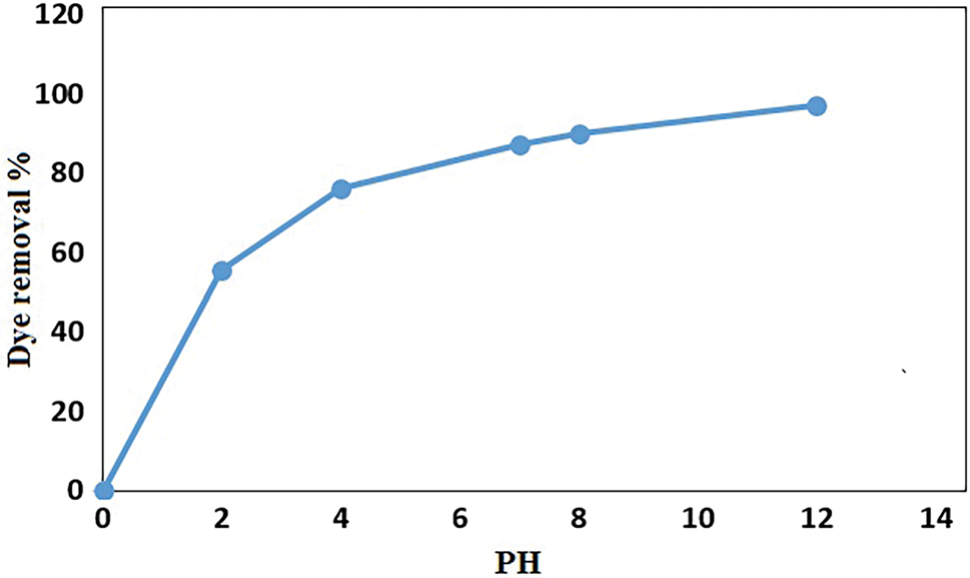
Effect of pH solution on removal of MB by GO/DETA/MnFe_2_O_4_@SiO_2_ composite (200 rpm, 25 °C, 10 min).

The pH of solution is an important factor due to separation of different functional groups on the adsorbent and ionization of adsorbent in solution. The effect of pH on the sorption of MB onto GO/DETA/MnFe_2_O_4_@SiO_2_ composite was investigated within pH range 2–12 (Fig. 11). As it can be observed in Fig. 11 with increasing the pH solution, the removal of MB was increased. At higher pH, the surface has a negative charge and produces electrostatic interactions with MB cationic molecules^50^.

### Adsorption isotherms

**Figure 12:**
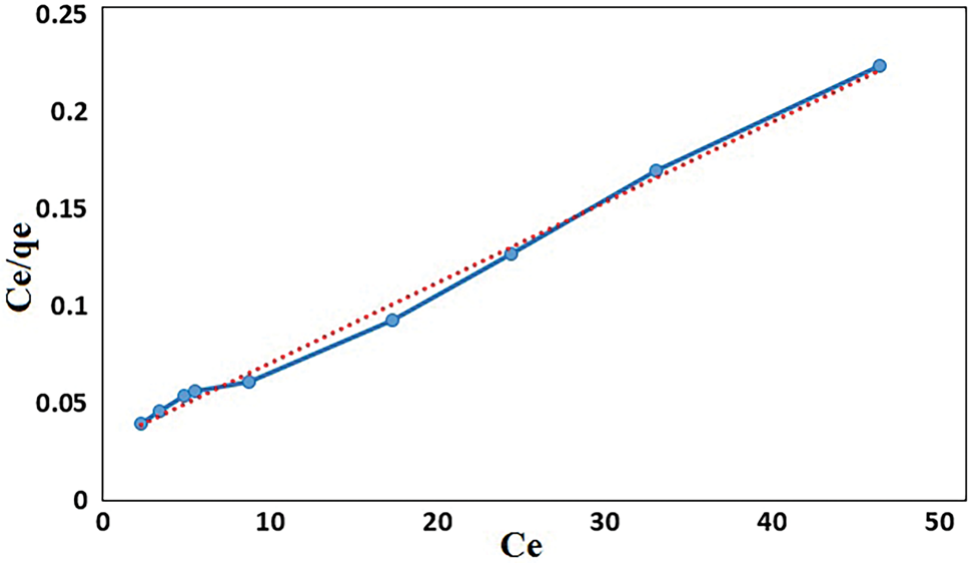
Langmuir plot for the adsorption of MB (200 rpm, 25 °C and pH = 7).

One of the important parameters for study of the interaction between adsorbent and dye are adsorption isotherms^51^. In this study, Langmuir and Freundlich isotherm models were applied to gain the isotherm parameters for adsorption of MB onto GO/DETA/MnFe_2_O_4_@SiO_2_ composite. One of the well-known models to study the monolayer adsorption is Langmuir isotherm model. In this model the surface contains finite number of sites having equal activation energy of adsorption. The linear model of Langmuir isotherm is showed as follows:

1 $$\frac{{{\text{C}}_{{\text{e}}} }}{{{\text{q}}_{{\text{e}}} }} = \frac{1}{{{\text{K}}_{{\text{L}}} \cdot {\text{q}}_{{\text{m}}} }} + \frac{{{\text{C}}_{{\text{e}}} }}{{{\text{q}}_{{\text{m}}} }}$$

where C_e_ is the concentration of the dye solution at equilibrium (mg/l), q_e_ is the maximum amount of dye adsorbed (mg/g), q_m_ indicates the value of monolayer adsorption capacity and K_L_ is the constant value of Langmuir (mg/l). The values of K_L_ and q_m_ were obtained from the plot of (C_e_/q_e_) versus C_e_. The Langmuir plot for the adsorption of MB onto GO/DETA/MnFe_2_O_4_@SiO_2_ composite is illustrated in Fig. 12.

**Figure 13:**
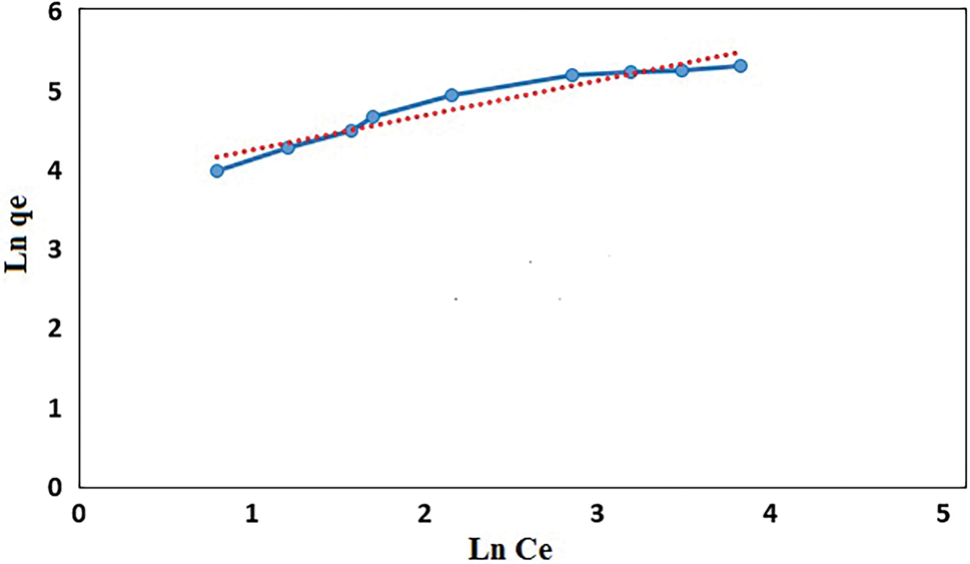
Freundlich plot for the adsorption of MB (200 rpm, 25 °C and pH = 7).

Freundlich isotherm model is used for reversible heterogeneous surface and is showed by the following linearized equation:

2 $${\text{Ln }}q_{e} = {\text{Ln K}}_{{\text{f}}} + \left( {\frac{1}{{\text{n}}}} \right) {\text{Ln C}}_{{\text{e}}}$$

where K_F_ and n are adsorption capacity (L/mg) and intensity of adsorption, respectively. The K_F_ and 1/n can be calculated from the linear plot of Ln qe versus Ln Ce (Fig. 13). The 1/n values represent irreversible (1/n = 0), favorable (0 < 1/n < 1) or unfavorable (1/n > 1) condition for adsorption.

The separation factor (R_L_) was determined by the following equation:

3 $${\text{R}}_{{\text{L}}} = \frac{1}{{1 + {\text{K}}_{{\text{L}}} \cdot {\text{C}}_{0} }}$$

where K_L_ is the Langmuir constant and C_0_ is the highest initial concentration of adsorbent (mg/l). The values of R_L_ can demonstrate the shape of the isotherm. If R_L_ > 1, the adsorption is unfavorable; if R_L_ = 1, the adsorption is linear; if 0 < R_L_ < 1, the adsorption is favorable and if R_L_ = 0 the adsorption is irreversible. Table 1 shows the values of Langmuir and Freundlich parameters and the regression coefficients (R^2^) of the MB onto GO/DETA/MnFe_2_O_4_@SiO_2_ composite. Based on R^2^ values, the experimental data were found to fit with Langmuir isotherm model for adsorbent. Also, the value of R_L_ lying in the range 0 < R_L_ < 1 confirms the favorable condition for adsorption of MB onto GO/DETA/MnFe_2_O_4_@SiO_2_ composite. Based on the result, the Langmuir model suggests homogeneous surfaces of the GO/DETA/MnFe_2_O_4_@SiO_2_ composite and monolayer coverage of MB onto the adsorbent. The maximum monolayer adsorption capacity (qm) was 243.91 mg/g for MB.

**Table 1:** Langmuir and Freundlich isotherms parameters and correlation coefficients for the adsorption of MB onto GO/DETA/MnFe_2_O_4_@SiO_2_ composite.

Langmuir isotherm parameters				Freundlich isotherm parameters		
q_m_ (mg/g)	K_L_ (L/mg)	R_L_	R^2^	K_F_ (L/mg)	n	R^2^
243.91	0.136	0.068	0.9951	44.45	2.29	0.9269

### Kinetic model for MB adsorption

**Figure 14:**
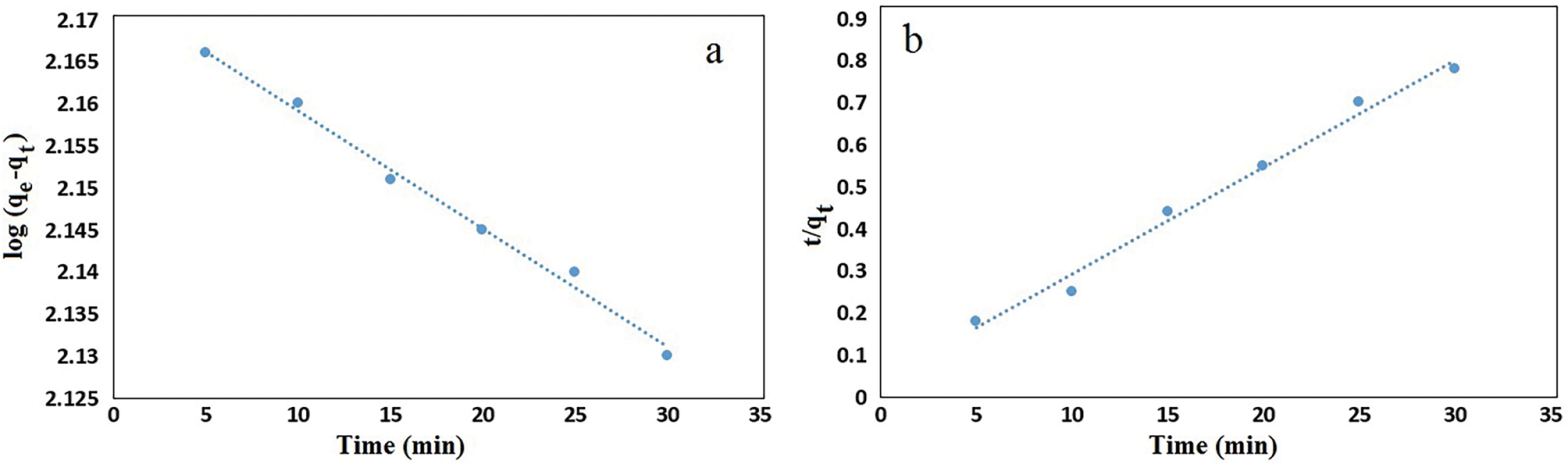
Pseudo-first-order (**a**) and Pseudo-Second-order (**b**) model for the removal kinetics of MB on GO/DETA/MnFe_2_O_4_@SiO_2_ composite (100 mg/l, 25 °C and pH = 7).

For investigating the mechanism of adsorption of MB two kinetic models: (i) pseudo-first order and (ii) pseudo-second order models have been studied. The linear form of pseudo-first-order^52^ and pseudo-second-order^53^ kinetic are expressed in Eqs. (4) and (5), respectively:

4 $$\log \left( {{\text{q}}_{{\text{e}}} - {\text{q}}_{{\text{t}}} } \right) = {\text{logq}}_{{\text{e}}} - \frac{{{\text{K}}_{1} }}{2.303}{\text{t}}$$

5 $$\frac{{\text{t}}}{{{\text{q}}_{{\text{t}}} }} = \frac{1}{{{\text{K}}_{2} {\text{q}}_{{\text{e}}}^{2} }} + \frac{{\text{t}}}{{{\text{q}}_{{\text{e}}} }}$$

where q_e_ and q_t_ (mg/g) is the amount of dye adsorbed at equilibrium and at time t, K_1_ and K_2_ (min^−1^) are the rate constants. The adsorption kinetics plots obtained for MB on GO/DETA/MnFe_2_O_4_@SiO_2_ composite are demonstrated in Fig. 14. The constants obtained for pseudo-first order and pseudo-second order models are listed in Table 2. Based on the results, the pseudo-first order model fit demonstrates a higher R^2^ value compared to the pseudo-second order for adsorbent. Also, the q_e_ value gained by calculating pseudo-first order kinetic is closer to the experimental value (177.25 mg/g).

**Table 2:** Kinetic parameters for the adsorption of MB onto GO/DETA/MnFe_2_O_4_@SiO_2_ composite.

Pseudo-first-order			Pseudo-Second-order		
q_e_ (mg/g)	K_1_ (× 10^–3^)(min^−1^)	R^2^	q_e_ (mg/g)	K_2_ (× 10^–3^)(min^−1^)	R^2^
147.91	3.22	0.9922	39.21	17.43	0.9876

### Thermodynamics of adsorption

**Figure 15:**
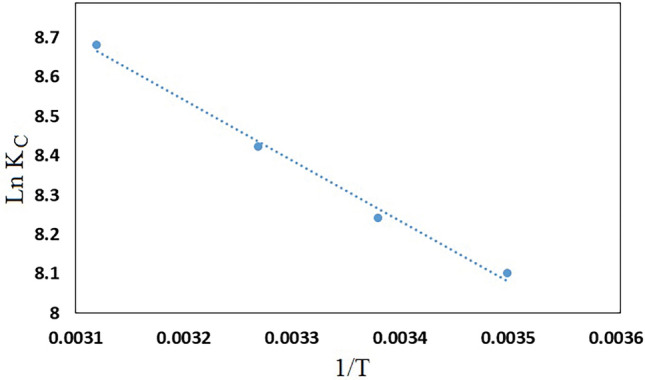
Thermodynamic plot for removal of MB on GO/DETA/MnFe_2_O_4_@SiO_2_ composite (200 rpm, 25 °C and pH = 7).

In order to study the thermodynamic factors such as standard enthalpy change (ΔH^°^), standard Gibbs free energy change (ΔG°) and standard entropy change (ΔS^°^), adsorption studies have been done at different temperatures (285–320 K). The thermodynamic factors were examined using following equations:

6 $${\text{Ln}} {\text{K}}_{{\text{C}}} = - \frac{{\Delta {\text{H}}^\circ }}{{{\text{RT}}}} + \frac{\Delta S^\circ }{R}$$

7 $$\Delta {\text{G}}^\circ = \Delta {\text{H}}^\circ - {\text{T}}\Delta {\text{S}}^\circ$$

where K_C_ is the thermodynamic equilibrium constant, T is the solution temperature and R is the universal gas constant (8.314 J/mol K). The values of $\Delta {\text{H}}^\circ$ and $\Delta {\text{S}}^\circ$ were determined from slope and intercept of plot Ln K_L_ vs 1/T (Fig. 15). Table 3 demonstrates the values of various thermodynamic parameters for the adsorption of MB on GO/DETA/MnFe_2_O_4_@SiO_2_ composite. The positive value of $\Delta {\text{H}}^\circ$ indicates that the adsorption of MB on GO/DETA/MnFe_2_O_4_@SiO_2_ composite is endothermic and the positive value of $\Delta {\text{S}}^\circ$ suggests the increase in randomness and disorder at the adsorbent-solution interface during the adsorption of MB on GO/DETA/MnFe_2_O_4_@SiO_2_ composite. Also, the negative values of $\Delta {\text{G}}^\circ$ show that the adsorption of MB on GO/DETA/MnFe_2_O_4_@SiO_2_ composite is spontaneous process.

**Table 3:** Thermodynamic parameters for the adsorption of MB on GO/DETA/MnFe_2_O_4_@SiO_2_ composite.

Adsorbent	Adsorbate	ΔH^°^ (kJ/mol)	ΔS^°^ (kJ/mol)	ΔG° (kJ/mol)			
				285 K	295 K	305 K	320 K
GO/DETA/MnFe_2_O_4_@SiO_2_	MB	12.53	0.12	− 21.67	− 22.87	− 24.07	− 25.87

### Adsorption mechanism

**Figure 16:**
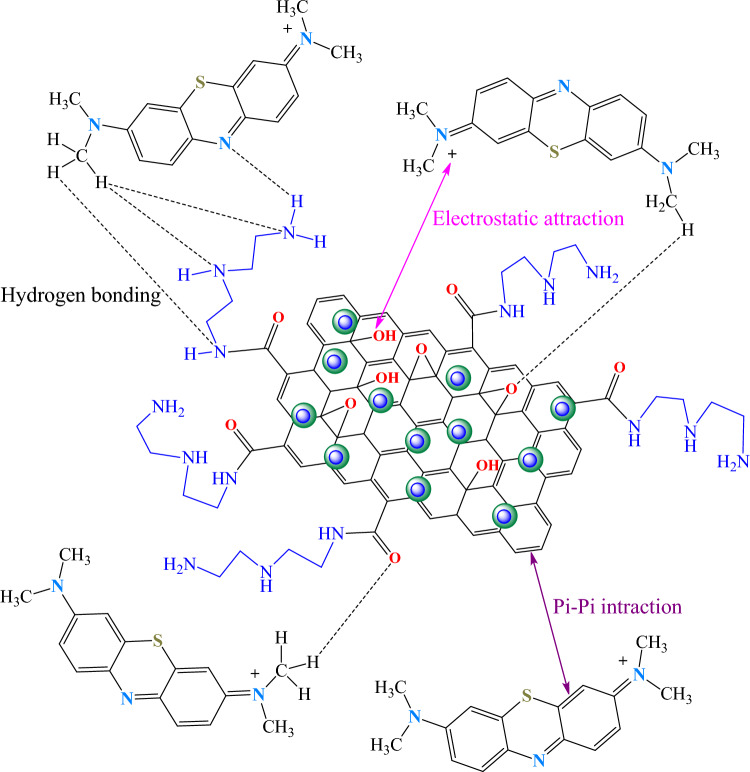
Different adsorption mechanisms of GO/DETA/MnFe_2_O_4_@SiO_2_ composite for MB.

The pH of solution plays an important role because of the separation of different functional groups on the adsorbent and ionization of adsorbent in solution. It was found that the adsorption efficiency increased with increasing pH (Fig. 11). With the increase of pH, the negative charges in the solution are increased. This could be because of the ionization of functional groups such as hydroxyl on the composite. Therefore, increasing the surface charge density raises the MB removal percentage due to the increasing electrostatic interactions between the negative charge of the composite and the positive charge of the MB (Fig. 16)^54^. As a result, the electrostatic interactions between the positive charge on nitrogen group of MB and the negative charge on oxygen group may act as the critical adsorption mechanism^55^. Also, the localized π electrons in the conjugated aromatic rings of the composite can interact by the C–C double bond of MB through π-π interaction^56^. Finally, the hydrogen bonding interactions between amine or oxygen containing groups of composite and hydrogen containing groups of MB play important role in the adsorption of MB on GO/DETA/MnFe_2_O_4_@SiO_2_ composite^57^ (Fig. 16).

### Reusability studies

**Figure 17:**
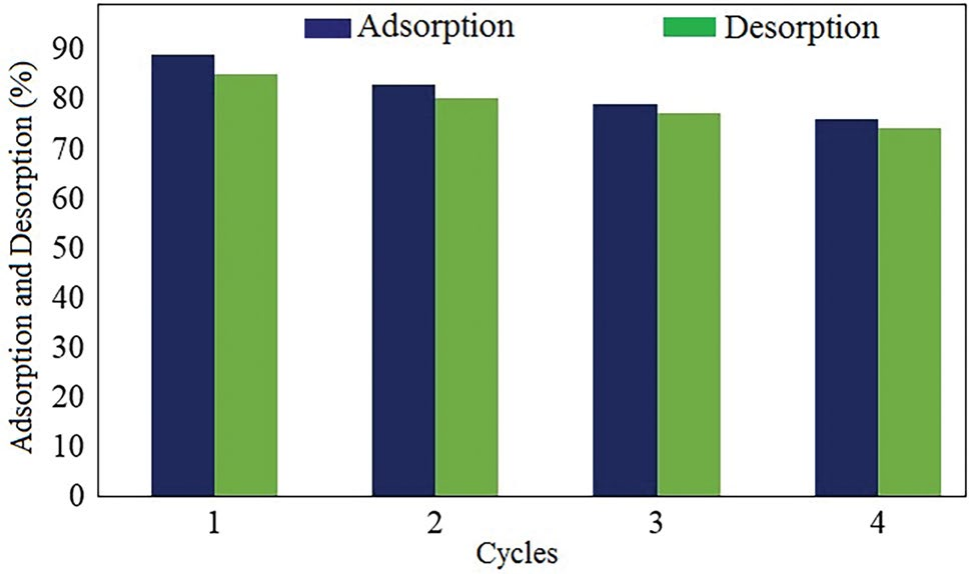
Regeneration studies for the adsorption–desorption of MB onto GO/DETA/MnFe_2_O_4_@SiO_2_ composite.

In the study of adsorption process, reusability is of great importance from the cost point of view in water treatment. To study the regeneration ability of the GO/DETA/MnFe_2_O_4_@SiO_2_ composite, four cycles of MB removal were evaluated^58^. The percentage removal of MB in 0.1 M HCl solution is represented in Fig. 17. As it can be observed from Fig. 17, the MB removal percentage decreased slightly and was still 74%.

### Comparison with other reported adsorbents

The adsorption capacity of GO/DETA/MnFe_2_O_4_@SiO_2_ composite for removal of MB was compared with GO-based adsorbents and other adsorbents reported in the literature (Table 4). As it is shown in Table 4, the adsorption capacity of GO/DETA/MnFe_2_O_4_@SiO_2_ composite is acceptable for removal of MB from aqueous solutions.

**Table 4:** Comparison of the adsorption capacity of present system with other reported systems.

Adsorbents	Dye	q_m_ (mg/g)	References
Graphene oxide/calcium alginate	MB	182	^59^
Graphene–carbon nanotube	MB	82	^60^
RCE/GO	MB	68	^61^
Pt–Co@GO	MB	90	^62^
GO/silicates	MB	90	^63^
GO-PDA-PSPSH	MB	185	^64^
GO	MB	100	^65^
Poly(AA-co-AMPS)/montmorillonite	MB	192	^66^
MgAl-layered double hydroxides	MB	102	^67^
Poly(AAc)	MB	220	^68^
Cu-Z-GO-M	MB	94.48	^69^
GO/DETA/MnFe_2_O_4_@SiO_2_	MB	243.91	Present study

## Conclusion

The GO/DETA/MnFe_2_O_4_@SiO_2_ composite has been successfully prepared and characterized by FTIR, XRD, SEM, EDS, VSM and TGA techniques. The composite showed magnetic property with a saturation magnetization of 3 emu/g. It was found that the GO/DETA/MnFe_2_O_4_@SiO_2_ composite was effective in adsorption of MB from aqueous solution. The effects of different parameters such as adsorbent dosage, initial drug concentration, pH and contact time were investigated. The Langmuir isotherm model was the best model to understand the adsorption process. According to the Langmuir analysis, the maximum adsorption capacity (qm) of the adsorbent for MB was obtained to be 243.91 mg/g. Kinetic studies demonstrated that the adsorption process followed pseudo-first order model for MB. The thermodynamic study illustrated that adsorption of MB on composite was spontaneous and endothermic, which was proceeded via hydrogen bonding, electrostatic and π-π interactions.

## Experimental

### Chemicals and instrumentation

All the chemicals were purchased from Merck. Methylene blue (chemical formula = C_16_H_18_ClN_3_S, Molecular weight (g/mol) = 319.85) was purchased from the Textile Factory. FT-IR spectra (Shimadzu prestige-21) were used to determine the identity of the as prepared nanocomposite. X-ray powder diffraction measurements were performed using an X-ray diffractometer (XRD) (Perkin Elmer) at ambient temperature. The surface morphology of the synthesized compounds was identified with a scanning electron microscope (LECO SEM, Michigan, USA). The elemental analysis was performed using energy-dispersive X-ray spectroscopy (EDS) on a scanning electron microscope, Mira 3-XMU model. Magnetic measurements were performed by means of the vibrating sample magnetometery method, using a VSM 7407 magnetometer, at room temperature. Thermogravimetric analysis (TGA) was performed using a Perkin Elmer thermogravimetric analyzer. UV–visible spectra in the 200–1000 nm range were obtained in DMF solvent on a Perkin Elmer Lambda 45 spectrophotometer. A Jenway model 4510 pH-meter was used for pH measurements by use of a combined electrode. An ultrasonication probe (Karl Deutsch, Germany) was used to disperse the nanoparticles in the solution.

### Preparation of the GO/DETA/MnFe_2_O_4_@SiO_2_ composite

#### Preparation of GO

GO was prepared using the reported modified method^70^. Graphite powder (2.0 g) were dissolved in a mixture of H_2_SO_4_ and H_3_PO_4_ (150:15) with stirring at 60 °C. Then, 10 g of KMnO_4_ was added to the mixture and stirred for 12 h until its color changed to brown. Afterward, the mixture was cooled then 300 ml deionized water and 4 ml H_2_O_2_ (30%) was added. The mixture was centrifuged and the residue was washed with HCl (10%) followed by deionized water several times until the pH achieved neutral and dried under vacuum.

#### Preparation of Manganese Ferrite Nanoparticles (MnFe_2_O_4_)

MnFe_2_O_4_ NPs was prepared using the reported modified method^71^. Briefly, 0.9 g of FeCl_3_.6H_2_O and 1.5 g of MnSO_4_·H_2_O were dissolved in 200 ml deionized water with stirring at 80 °C. Then, NaOH (8 M) was added slowly to the solution to raise the pH to 10. The solution was stirred under nitrogen gas at 80 °C for 3 h. Afterward, MnFe_2_O_4_ NPs precipitates was separated by a magnetic separation and then washed with deionized water and ethanol. Then, the MnFe_2_O_4_ NPs were dried at 60 °C for 24 h.

#### Preparation of silica-coated nanoparticles MnFe_2_O_4_@SiO_2_

MnFe_2_O_4_ (6 g) was dispersed in a 100 ml of 0.1 M HCl aqueous solution. Then, solution ultrasonically agitated for 20 min. The nanoparticles isolated and washed with deionized water. Then, the nanoparticles were suspended 40 ml deionized water, 100 ml ethanol and 15 ml 28% ammonia solution and stirred for 1 h. Afterward, tetraethylorthosilicate (20 ml) in 20 ml ethanol was added to solution. This solution was stirred for 10 h at room temperature. Then, the product MnFe_2_O_4_@SiO_2_ were separated by an external magnet and washed with deionized water and ethanol.

#### Preparation of GO/DETA/MnFe_2_O_4_@SiO_2_ composite

0.5 g of GO was placed into 100 ml DMF and ultrasonically dispersed for 1 h. Subsequently, 0.5 g MnFe_2_O_4_@SiO_2_ and 70 ml Diethylenetriamine (DETA) were added into to flask. The mixture refluxed with stirring for 24 h. the composite was isolated and washed with ethanol and dried in a vacuum oven at 50 °C.

### Adsorption experiments

GO/DETA/MnFe_2_O_4_@SiO_2_ composite was used for removal of MB dye from aqueous solutions. For this aim, different factors such as adsorbent dose, contact time, initial concentration and PH on adsorption were investigated. For doing the experiments, solution of 100 mg/l of MB was prepared in deionized water. To prepare the experimental solutions, different amounts of composite (5–25 mg) were placed in a series of 40 ml of dye solution with different concentrations (40–120 mg/l) in 50 ml glass flasks. To study the effect of contact time of adsorbent on MB, prepared suspension solutions were stirred for 10–20 min. Beside, by using solutions of 0.01 N HCl or NaOH the effect of pH on the amount of adsorption was studied. The concentration of the MB was calculated by UV-spectrophotometer at λ_max_ = 600 nm. Equations (8) and (9) were used to calculate the amount of dye adsorbed on the adsorbent (qe in mg/g) and the percentage of solution dye removal (R in %):

8 $${\text{qe}} = \frac{{\left( {{\text{C}}0 - {\text{Ce}}} \right)}}{{\text{M}}} \cdot {\text{V}}$$

9 $${\text{\% R}} = \frac{{\left( {{\text{C}}0 - {\text{Ce}}} \right)}}{{{\text{C}}0}} \cdot 100$$

where C_0_ and C_e_ are the initial and equilibrium concentration of dye in solution (mg/l), respectively. V is the initial volume of the dye solution (l) and M is the mass of adsorbent used (g).

## Data Availability

All data generated or analyzed during this study are included in this published article.
